# Verapamil induces autophagy to improve liver regeneration in non-alcoholic fatty liver mice

**DOI:** 10.1080/21623945.2021.1983241

**Published:** 2021-10-26

**Authors:** Jian-Lin Lai, Yuan-E Lian, Jun-Yi Wu, Yao-Dong Wang, Yan-Nan Bai

**Affiliations:** a Shengli Clinical Medical College of Fujian Medical University, Department of Hepatobiliary and Pancreatic Surgery, Fujian Provincial Hospital, Fuzhou, Fujian, 350001, China; b Department of Pathology, The Affiliated Union Hospital of Fujian Medical University, Fuzhou, 350001, China

**Keywords:** Verapamil, autophagy, non-alcoholic fatty liver disease, liver regeneration

## Abstract

Verapamil can restore intracellular calcium homeostasis, increase the fusion of autophagosomes and lysosomes, reduce lipid droplet accumulation and inhibit inflammation and insulin resistance in high-fat-fed mice. The present study aimed to investigate verapamil's effect and its underlying liver regeneration mechanism in mice with non-alcoholic fatty liver. After 50% hepatectomy was performed, the changes of autophagy and liver regeneration were evaluated by detecting cell proliferation and autophagy at each time point. Then, 25mg/kg verapamil was injected intraperitoneally for 10 d before an operation in the mild to moderate fatty liver and severe fatty liver groups. The control group and mild to moderate fatty liver group reached the peak of proliferation at 24-48h after operation, and the mice with severe fatty liver and steatohepatitis reached the peak at 48-72h. Autophagy in the normal group and mild to moderate fatty liver group reached the peak 48 hours after operation. Verapamil injection can enhance autophagy, reduce the weight of fatty liver mice, improve liver function and liver regeneration. Verapamil can induce autophagy, improve hepatocyte function and promote hepatocyte regeneration through the mTOR independent signaling pathway, thus improving the process of liver regeneration after partial hepatectomy.

## 1. Introduction

Non-alcoholic fatty liver disease (NAFLD) is a clinical syndrome characterized by hepatic parenchymal cell steatosis, intralobular inflammation, and mild fibrosis without previous history of alcoholism while excluding other causes. The pathological types include simple non-alcoholic fatty liver (NAFL), non-alcoholic steatohepatitis (NASH), liver fibrosis, and liver cirrhosis,which can develop into liver cancer in severe cases [1,2]. The pathogenesis of NAFLD is complex. At present, most people agree with the theory of ‘two strikes’ put forward by Day and James [3], that is, the first blow is simple hepatocyte steatosis caused by hyperinsulinemia and insulin resistance, and the second blow is oxidative stress, lipid peroxidation, mitochondrial dysfunction, inflammatory cytokine release, and other factors to induce liver inflammation, hepatocyte degeneration and necrosis, liver fibrosis and cirrhosis. In recent years, it has been reported [4–8] that hepatic steatosis increases the complications and mortality of liver surgery, which is an independent risk factor for the prognosis of liver surgery, especially for major hepatectomy, and different degrees of fatty liver have different effects on the safety of liver surgery.

Previous studies have shown that autophagy can degrade lipid droplets in hepatocytes, improving the fatty liver, a process known as lipophagy [9,10]. Early fat accumulation and hepatocyte steatosis are the basis of NAFLD. Autophagy can provide energy for life activities by phagocytosis of lipid droplets in hepatocytes during starvation. In a high-fat diet, autophagy can degrade lipids and reduce intrahepatic fat accumulation and hepatocyte steatosis. When autophagy is inhibited, the condition of NAFLD is aggravated [11]. Therefore, autophagy can be used as an important research focus in the treatment of NAFLD. Enhanced autophagy can improve insulin sensitivity, reduce lipid deposition in hepatocytes, reduce cell damage caused by cytokines and oxidants, inhibit macrophage activation, and reduce inflammation, to prevent apoptosis and protect hepatocytes.

Verapamil (VER) is an L-type calcium channel blocker, which can reduce intracellular calcium overload. Verapamil has been widely used in the treatment of hypertension and arrhythmia. Park et al. found that calcium overload in hepatocytes of mice fed with high fat inhibited the fusion of autophagosomes and lysosomes, resulting in reduced autophagy flow, which was not conducive to lipid phagocytosis [12]. Then, verapamil can restore intracellular calcium homoeostasis, increase the fusion of autophagosomes and lysosomes, reduce lipid droplet accumulation and inhibit inflammation and insulin resistance [13]. Verapamil can enhance autophagy and improve fatty liver function, but whether verapamil can enhance autophagy to promote liver regeneration has not been reported. This study aimed to investigate whether verapamil can promote liver regeneration by inducing autophagy and improving the fatty liver’s function.

## 2. Materials and methods

### 2.1. Grouping of experimental animals and animal models

Male C57BL/6 mice were purchased from Shanghai SLAC Laboratory Animal Company. After one week of adaptive feeding, 5-week-old healthy mice were randomly divided into normal group, mild to the moderately fatty liver group, severe fatty liver group, and steatohepatitis group. All the animals were raised in the Animal Experimental Center of Fujian Medical University. The laboratory’s constant temperature was 22 ± 1°C, the relative humidity was 55 ± 5%, and the cycle of day and night was 12 h. Before hepatectomy, there was no food or drink for 12 h. All the experiments were carried out following the ethical review requirements of animal experiments at Fujian Medical University.

Mice in the control group were fed with D12450B forage for 4 months. The fatty liver groups were fed with a high-fat diet D12490 for different times, the mild to the moderately fatty liver group was fed for 2 months, and the severe fatty liver group was fed for 4 months. After 4 months, the steatohepatitis group was intraperitoneally injected with 20% carbon tetrachloride (CCl4, 0.5 ml per kg body weight) twice a week for 2 weeks. Finally, the model’s success was confirmed by mouse body weight, liver tissue HE staining, and oil red O staining, according to the range of hepatocyte steatosis. Through semi-quantitative assessment of the degree of steatosis, 5%-33% were mild steatosis, 33%-66% were moderate steatosis, and more than 66% were severe steatosis. Ballooning degeneration and intralobular inflammation of the liver were steatohepatitis. They were divided into a normal group (ND4m), mild to moderately fatty liver group (HFD2m), severe fatty liver group (HFD4m), and fatty hepatitis group (HFD+CCl4). Then, the mild to moderately fatty liver andsevere fatty liver groups were intraperitoneally injected with verapamil (25 mg per kg body-weight for 10 d), while the control group was injected with the same dose of normal saline, which was divided into HFD2m+NS, HFD2m+VER, HFD4m+NS, and HFD4m+VER.

### 2.2. 50% hepatectomy model(Partial hepatectomy, PH)

After establishing the fatty liver model, 50% hepatectomy was performed to establish the liver regeneration model. Surgery was performed under 2% isoflurane continuous inhalation anaesthesia. According to Higgins’s hepatectomy, the left lateral and right lobes (right upper lobe and right lower lobe) of the liver were resected.The mice were killed at 0, 12, 24, 36, 48, 72, 120, and 168 h after hepatectomy, blood was collected and centrifuged, and plasma stored at −80°C. Livers were removed, weighed, and thin slices of all livers lobes immersed in 10% formalin or snap-frozen immediately in liquid nitrogen for further analysis.

### 2.3. Detection of liver function and liver metabolic indexes

The body-weight of mice was detected by electronic balance before an operation or before drug injection in the morning. The detection of biochemical blood indexes showed that the amount of blood collected by eyeball puncture or eyeball extraction was about 0.5–1.0 ml before the operation, and at each time point after an operation, the blood was kept on ice for 30 min, and the upper serum was kept at −80°C after 10 min centrifuge at 8000 rpm/min with low temperature (−4°C). Glucose (GLU), alanine aminotransferase (ALT), aspartate aminotransferase (AST) were detected by rate method on an automatic biochemical analyser. The oxidative stress reactive oxygen species (ROS) index was detected when the liver tissue was cut off at different time points after an operation and preserved at −80°C. During the detection, the liver tissue homogenate was prepared according to the instructions, and then malondialdehyde (MDA), superoxide dismutase (SOD), peroxidase (POD), and GLUtathione (GSH) were detected according to the ROS detection kit manual.

### 2.4. Detection of liver regeneration indexes

The resected and residual livers’ weight was weighed at 0 (operation), 6, 12, 24, 48, 72, 120, and 168 h after PH, respectively.The liver’s coefficient to body weight was calculated as the ratio of wet liver weight to body weight. Liver regeneration rate was expressed as a percentage of regenerated liver mass calculated using the equation [C-(A-B)]/A, where A was the estimated total liver mass at the time of liver resection, B was the resected liver’s wet weight,and C was the residual liver mass regenerated at the corresponding time. Proliferating cell nuclear antigen (PCNA) expression was also used to evaluate liver, according to the immunohistochemical staining steps to detect hepatocyte PCNA quantify. One hour prior to sacrifice at different time points after PH, a single injection of 10 mg/ml BrdU (5-bromo-2ʹ-deoxyuridine) was administered intraperitoneally at a dose of 100 mg/kg animal weight. Liver tissue BrdU was detected by immunohistochemistry and quantified by the same method as above.

### 2.5. Detection of autophagy index

Western blotting (WB): after hepatectomy, the liver tissue was stored at −80°C. The regenerated liver tissue of each group was ground with liquid nitrogen.The liver tissue was dissociated with proteolysis solution, the total protein of liver tissue was extracted.The protein content was determined. The supernatant was collected and added to the sample buffer, boiled in boiling water at 100°C for 5 min, and stored at −20°C. Western blotting was used to detect the markers of autophagy-related proteins, such as LC3, p62, Beclin1, Atg7, mTOR, etc.

Immunofluorescence (IF): after hepatectomy, the liver tissue was stored at −80°C and transported at low temperature. After paraffin embedding, frozen sections were made and stored at −20°C. According to immunofluorescence imaging, the co-localization of LC3 and LAMP1 was observed to determine the binding of autophagosomes and lysosomes.

### 2.6. Examination by transmission electron microscope (TEM)

For TEM, liver biopsies were fixed in glutaraldehyde(3%) plus *p*-formaldehyde (1.5%) for several hours. Samples were postfixed in 1% osmium tetroxide for 90 min at 25°C, stained with uranyl acetate (5 mg/ml) for 1.5 h at 25°C, dehydrated in acetone and embedded. Ultra-thin sections, unstained or poststained with uranyl acetate and lead hydroxide, were examined under a TECNAI transmission electron microscope of FEI Company. The cellular structure, autophagosomes, and organelles such as mitochondria and lysosomes in the process of liver regeneration were observed.

### 2.7 Main reagents

High-fat diet D12490 and control diet D12450B were purchased from the American Diet-Research Company. Saturated Oil Red O dyeing solution was obtained from Beijing Solarbio Technology Co., Ltd. Malondialdehyde (MDA) kit A003-1, superoxide dismutase (SOD) kit A001-3, peroxidase (POD) kit A084-1, glutathione (GSH) test kit A006-1 were from Nanjing Jiancheng Institute of Biological Engineering.Verapamil hydrochloride (V4629) was purchased from Sigma-Aldrich of the United States. The membranes were immunoblotted with anti-LC3 (Abcam, ab19, Cambridge, UK), anti-Proliferating Cell Nuclear Antigen (PCNA) (Abcam, ab29, Cambridge, UK), anti-Beclin 1 (Abcam, ab207612, Cambridge, UK), anti-p62/SQSTM1 (Santa Cruz Biotechnology, Sc-28,359, Santa Cruz, CA, USA), anti-Atg7, anti-phosphorylated and total mTOR, anti-phosphorylated and total 4EBP1, anti-phosphorylated and total p70 S6 Kinase (Cell Signalling Technology, #2631, #4060, #9272, #2971, #2983, #9205, #2708, Beverly, MA, USA). Carbon tetrachloride (CCl4) and olive oil were from Shanghai Xinzhong Chemical Reagent Factory.

### 2.8 Statistical analysis

Experimental data are presented as the mean ±SD. The Student’s *t*-test (two-tailed) was used to compare the data. Multiple groups were analysed by one-way ANOVA with SPSS 20.0 software. Differences were considered statistically significant at *P*< 0.05.

## 3. Results

### 3.1. Fatty liver mouse model

**Figure 1.**
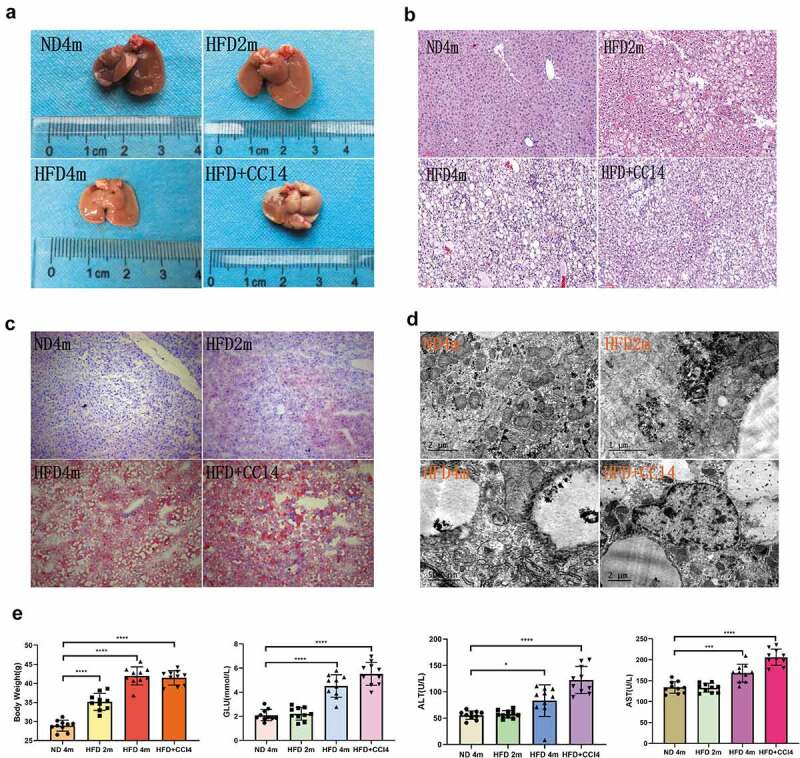
The mice with different degrees of fatty liver were successfully modelled. a: The general morphology of liver in the control group, mild to the moderately fatty liver group, severe fatty liver group, and steatohepatitis group; b: The degree of steatosis was observed by HE staining in four groups. c: Four groups of liver parenchyma Oil Red O staining; d. The hepatocytes’ ultrastructure was observed by TEM; E:Four groups of biochemical indexes. (*:0.01 < *P*< 0.05, **: 0.001 < *P*< 0.01, ***: 0.0001 < *P*< 0.001,****: *P*< 0.0001, n = 10, HE and Red O staining scale bar: 100 μm.)

The models of normal group (ND4m), mild to themoderately fatty liver group (HFD2m), severe fatty liver group (HFD4m), and fatty hepatitis group (HFD+CCl4) were established successfully. In general, the normal group’s liver showed smooth and ruddy, no white-spotted nodules, tougher texture, flexible and soft. With the increase of the degree of fatty liver, the liver’s surface colour gradually whitened, appeared white spot-like changes, and the texture became brittle (Figure 1(a)). In normal liver tissue, HE and Oil Red O staining showed that hepatocytes were arranged regularly. The hepatic sinusoid space was clear with no inflammatory cell infiltration; the morphology and size of hepatocytes were normal, there was no fat or balloon degeneration. With the aggravation of steatosis of the liver, the arrangement of hepatocytes was disordered, the cord-like structure gradually disappeared, the hepatocytes were enlarged, the cytoplasm was loose, a large number of lipid droplets were gathered in the cells (Figure 1(b)), and the vacuoles were stained red under oil red staining (Figure 1(c)). The nuclei were squeezed to the cytoplasmic margin in severe fatty liver, and fat droplets were found in more than 66% of cells. In the steatohepatitis group, inflammatory cells infiltrated in the hepatocytes’ intercellular space, some of them showed balloon-like changes, and no liver fibrosis was found. The ultrastructure of liver tissue observed by TEM (Figure 1(d)) showed that the hepatocytes in normal liver tissue were polygonal, the morphology and structure of organelles were normal, and the hepatic sinusoid space was normal.

In the steatohepatitis group, fat vacuoles of different sizes could be seen in the cytoplasm, especially around the nucleus; meanwhile, lysosomes and mitochondria were swollen and deformed, and some normal organelles disappeared. In biochemical indexes (Figure 1(e)), there was no significant difference in GLU, ALT, and AST between the control group and mild tomoderately fatty liver group. When reaching severe fatty liver and steatohepatitis, the body-weight of the fatty liver group was significantly higher than that of the control group, the indexes of ALT, AST, and GLU increased significantly.

### 3.2 Comparison of basic autophagy in resting-state among four groups and the changes of autophagy after hepatectomy

**Figure 2.**
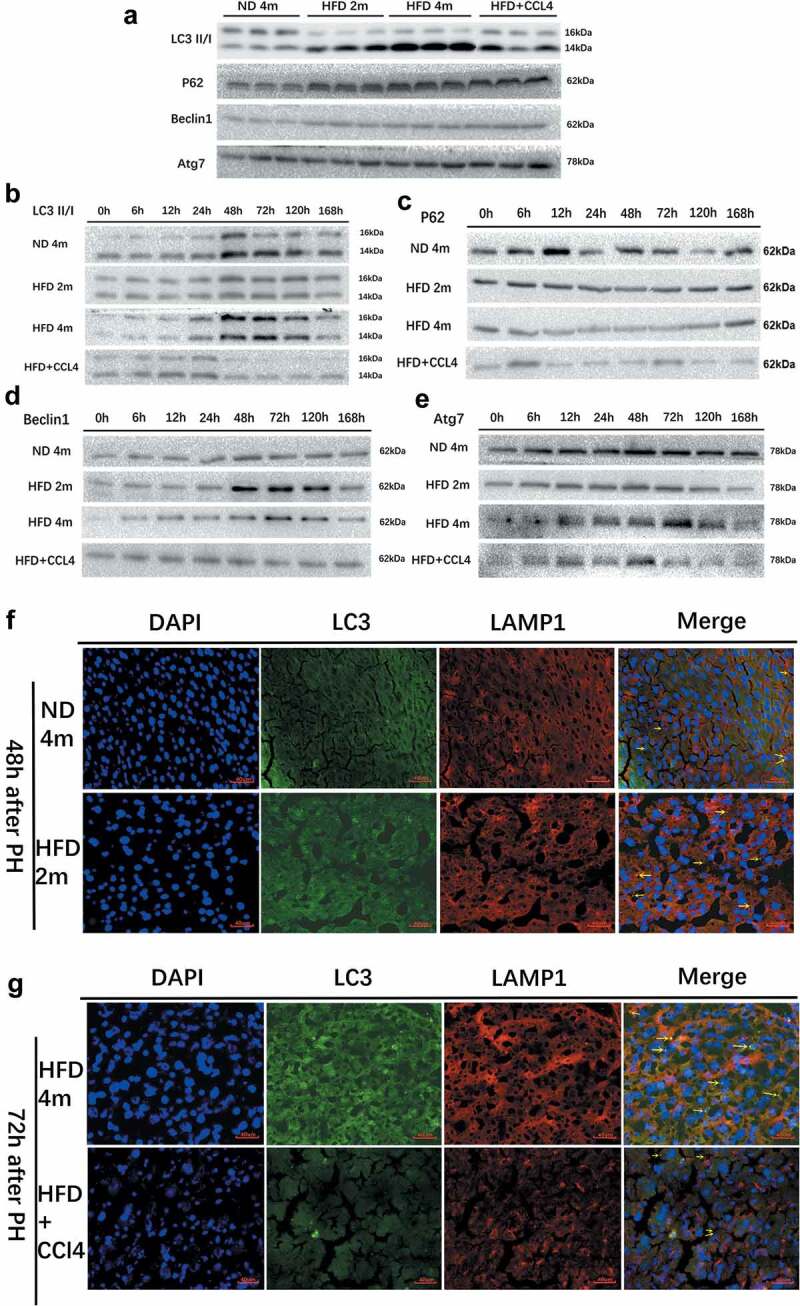
Autophagy was observed in the control group (ND4m), mild to moderately fatty liver group (HFD2m), severe fatty liver group (HFD4m), and fatty hepatitis group (HFD+CCl4) at rest and after hepatectomy. a: The autophagy state of the four groups in the resting state, WB detection of autophagy-related proteins LC3II/I, p62, Beclin1, Atg7; b: WB to detect the changing trend of LC3II/I of the four groups; c: WB detection of p62; d: WB detection of Beclin1; e: WB detection of Atg7; f: LC3 and LAMP1 immunofluorescence co-localization imaging of the ND4m and HFD2m after PH 48 h; g: LC3 and LAMP1 immunofluorescence co-localization imaging of the HFD4m and HFD+CCl4 after PH 72 h. The yellow arrows represented autophagolysosomes, scale bar: 40 μm

In the resting state, the autophagy level in the severe fatty liver group was the strongest, showing that the protein expression level of LC3II/I, Beclin1, and Atg7 was the highest (Figure 2(a), Supplementary Figure S1(a)). However, p62 protein also increased, indicating that the whole autophagy flux process was damaged, especially in steatohepatitis group. The level of autophagy was different among the steatohepatitis groups (Figure 2(a)). The autophagy level in the control group and mild to moderately fatty liver group peaked at about 48 h after PH, and the expression of LC3II/I, Beclin1, and Atg7 was the strongest at 48 h after PH. p62 protein had no obvious trend, and the level of autophagy decreased with time. The autophagy level in the severe fatty liver group peaked at 72 h after PH, while the overall autophagy level in the steatohepatitis group was weak and had no obvious trend (Figure 2(b-e), Supplementary Figure S1(b-e)). Moreover, the immunofluorescence co-localization of LC3 and LAMP1 showed that the positive rate of expression in the control group and mild to the moderately fatty liver group was the highest at 48 h after an operation and that in the severe fatty liver group was the highest at 72 h after an operation (Figure 2(f-g)). These results showed that the level of autophagy increases first and then decreases after hepatectomy.

### 3.3. Liver regeneration after hepatectomy in four groups: ND4m,HFD2m, HFD4m, HFD+CCl4

**Figure 3.**
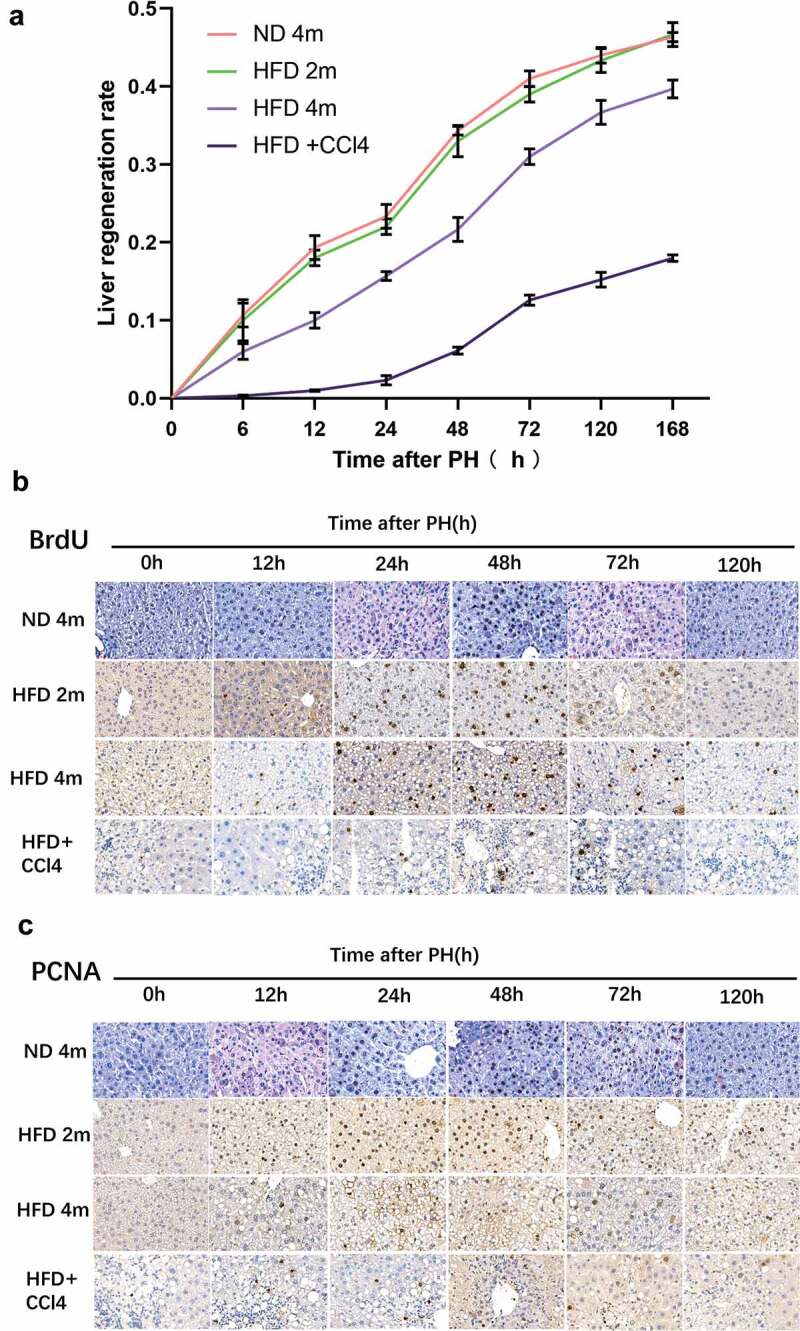
After 50% hepatectomy, liver regeneration was performed in the ND4m, HFD2m, HFD4m, and HFD+CCl4. a: The changing trend of liver regeneration rate in the four groups at 0, 6, 12, 24, 48, 72, 120, and 168 h after hepatectomy; b: The expression of BrdU which was detected by the immunohistochemical method after PH; c: The expression of PCNA after PH. Scale bar: 20 μm

The proliferation trend of the control group and mild to the moderately fatty liver group was roughly the same, starting at 12 h after surgery, the slope of the liver regeneration rate was the largest between 24–48 h after surgery (Figure 3(a)) combined with BrdU (Figure 3(b)) and PCNA (Figure 3(c)), while this period of liver regeneration was at the peak. The severe fatty liver group’s overall liver regeneration level was weaker than that of the control group and mild tothe moderately fatty liver group, and the peak of liver regeneration was 48–72 h after an operation. The liver regeneration process in the steatohepatitis group was weak, while liver regeneration peaked at 48–72 h after an operation.The above results revealed that liver regeneration increased first and then decreased after hepatectomy. The peak of liver regeneration in the fatty liver group was delayed. Moreover, the peak of liver regeneration in each group was earlier than that of autophagy.

### 3.4. Verapamil improve liver function and promote autophagy

**Figure 4.**
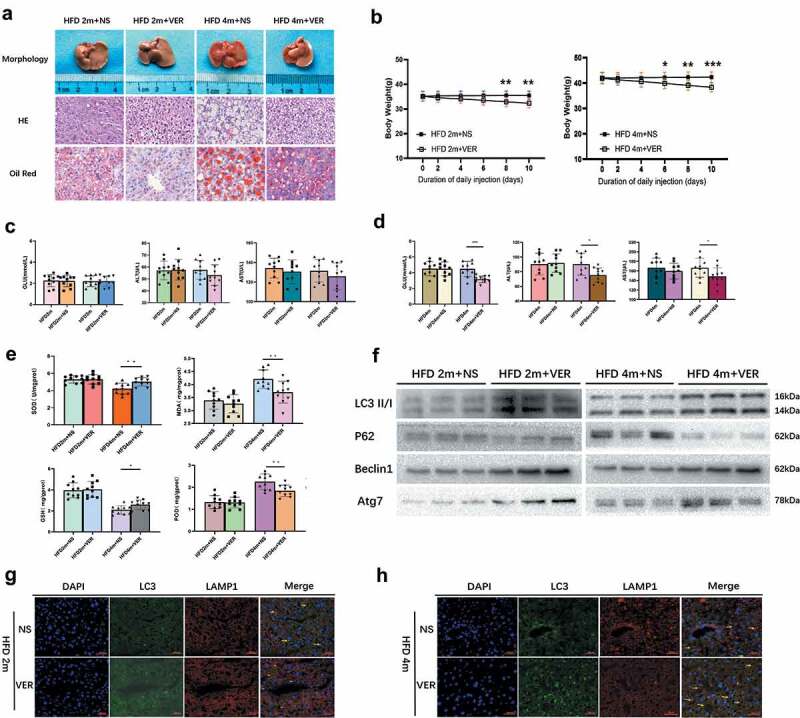
The changes in body weight, liver morphology, liver function, oxidative stress, and autophagy of mice in the HFD2m and HFD4m after intraperitoneal verapamil injection. a: After injection of verapamil 25 mg/kg for 10 d, the changes of general morphology, histological status, and oil red staining; b: The changes of body weight within 10 d after injection of verapamil; c: The changes of GLU, AST and AST in the HFD2m after verapamil injection; d: The changes of the HFD4m’s GLU, AST, and AST after verapamil injection; e: The changes of SOD, GSH, MDA, and POD after verapamil injection; f: Changes of autophagy-associated proteins in mice injected with verapamil; g, H: LC3 and LAMP1 IF co-localization imaging, the yellow arrows represented autophagolysosomes. *: 0.01 < *P*< 0.05, **: 0.001 < *P*< 0.01, ***: 0.0001 < *P*< 0.001, ****: *P*< 0.0001, n = 10, HE and Red Ostaining scale bar: 100 μm, IF scale bar: 40 μm

Injection of verapamil before hepatectomy in mild to themoderately fatty liver group (HFD2m) and severe fatty liver group (HFD4m) could reduce body weight, accelerate liver function, improve liver morphology and promote autophagy. Ten days after intraperitoneal injection of verapamil in the HFD2m and HFD4m, the liver’s naked eye morphology gradually turned ruddy. Fat vesicles decreased under HE and oil red staining (Figure 4(a)), and body weight also decreased significantly (Figure 4(b)). There was no significant decrease in GLU, ALT, and AST in the HFD2m after verapamil injection (Figure 4(c)). In the HFD4m, GLU, AST, and ALT decreased significantly after verapamil injection (Figure 4(d)).Simultaneously, SOD and GSH increased significantly, while MDA and POD decreased significantly (Figure 4(e)). After injection of verapamil, the expression of LC3II/I, Beclin1 and Atg7 increased, the expression of protein p62 decreased, and the co-localization expression of LC3 and LAMP1 increased, indicating that the formation of autophagy-lysosome increased and the whole process of autophagy enhanced (Figure 4(f-h)). These results suggested that verapamil can enhance autophagy and improve liver function.

### 3.5. Changes of autophagy after hepatectomy in the HFD2m and HFD4m after verapamil injection

**Figure 5.**
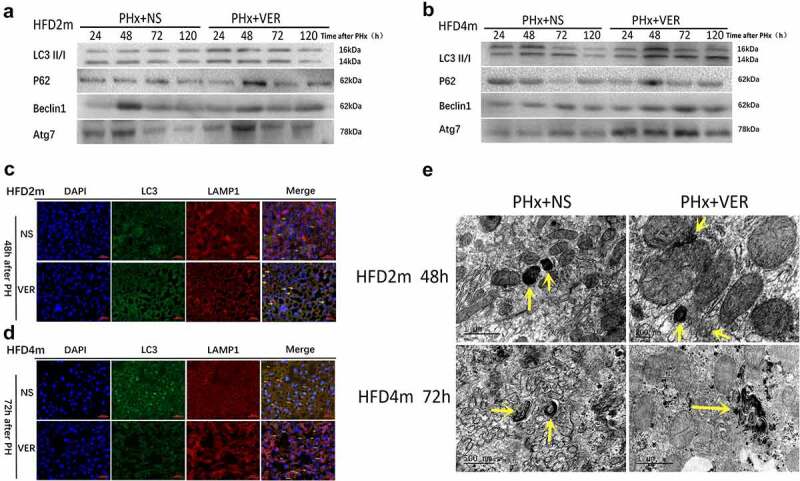
Autophagy changes of mice in HFD2m and HFD4m after 50% hepatectomy after injection of verapamil. a: Comparison of autophagy levels at 24, 48, 72, and 120 h after hepatectomy in HFD2m after injection of normal saline and verapamil respectively; b: Comparison of autophagy level after hepatectomy in HFD4m after injection of normal saline and verapamil; c, d: LC3 and LAMP1 IF co-localization imaging (48 h after hepatectomy in the HFD2m and 72 h after hepatectomy in the HFD4m, the yellow arrows represented autophagolysosomes, scale bar: 40 μm); e: Obvious autophagosomes were observed after hepatectomy, and the yellow arrows represented autophagosomes

In the HFD2m group, the level of autophagy increased after injection of verapamil, which showed that the expression of LC3II/I, Beclin1, and Atg7 in the experimental group increased at each time point after an operation, while the expression of p62 protein decreased as a whole, and autophagy reached the peak at 48 h after operation in both groups (Figure 5(a)). In the HFD4m group, the expression of LC3II/I, Beclin1 and Atg7 increased after injection of verapamil, but there was no significant difference in the expression of p62 protein, indicating that the level of autophagy increased and reached the peak of autophagy at 72 h after operation (Figure 5(b)). Moreover, the co-localization expression of LC3 and LAMP1 increased after verapamil injection in both groups (Figure 5(c,d)), indicating an increased level of autophagy. Under TEM, the formation of autophagosomes can be observed in the HFD2m group at 48 h after hepatectomy, and the formation of autophagosomes can be observed at 72 h in the HFD4m group (Figure 5(e)).

### 3.6. HFD2m and HFD4m received an injection of verapamil promoted liver regeneration after PH

**Figure 6.**
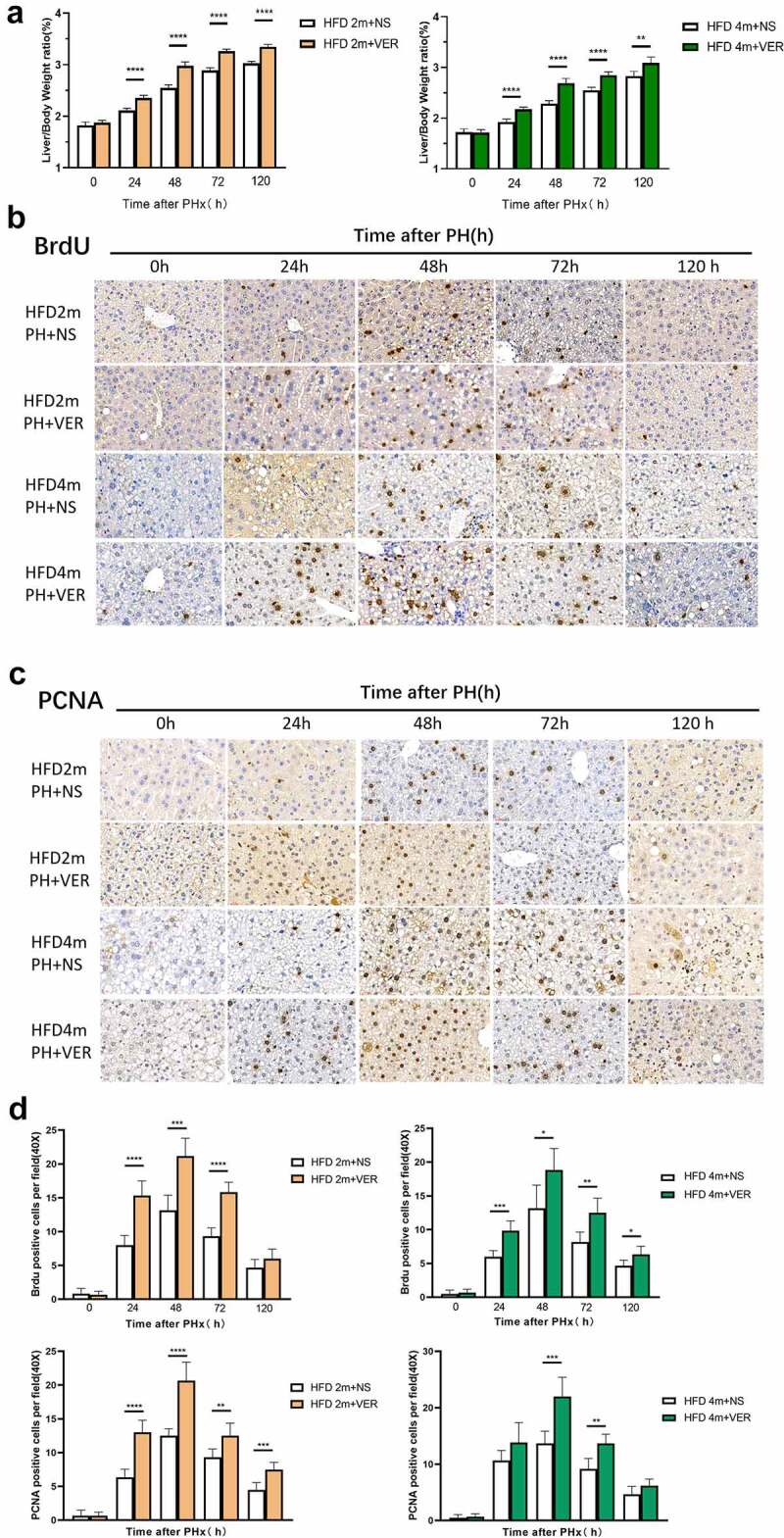
The HFD2m and HFD4m were injected with normal saline and verapamil, respectively, after hepatectomy. a: The changes of liver weight to body weight ratio at different time points after hepatectomy in the two groups after injection of normal saline and verapamil respectively; b: After injection of normal saline and verapamil, the expression of BrdU which was detected by the immunohistochemical method after PH; c: The expression of PCNA after PH; d: Quantitative results of BrdU and PCNA. (*: 0.01 < *P*< 0.05, **: 0.001 < *P*< 0.01, ***: 0.0001 < *P*< 0.001, ****: *P*< 0.0001, n = 10, scale bar: 20 μm.)

The ratio of liver weight to body weight in HFD2m and HFD4m at 24, 48, 72, and 120 h after an operation was significantly higher than that in the normal saline group (Figure 6(a)). The immunohistochemical results of BrdU and PCNA showed that the positive rate of nuclear staining in the verapamil group was higher than that in the normal saline group (Figure 6(b,c)), and the quantitative results (Figure 6(d)) suggested that the difference was statistically significant. These results suggested that liver regeneration can be improved after verapamil injection in HFD2m and HFD4m.

### 3.7. Verapamil induced autophagy through mTOR independent signal pathway

**Figure 7.**
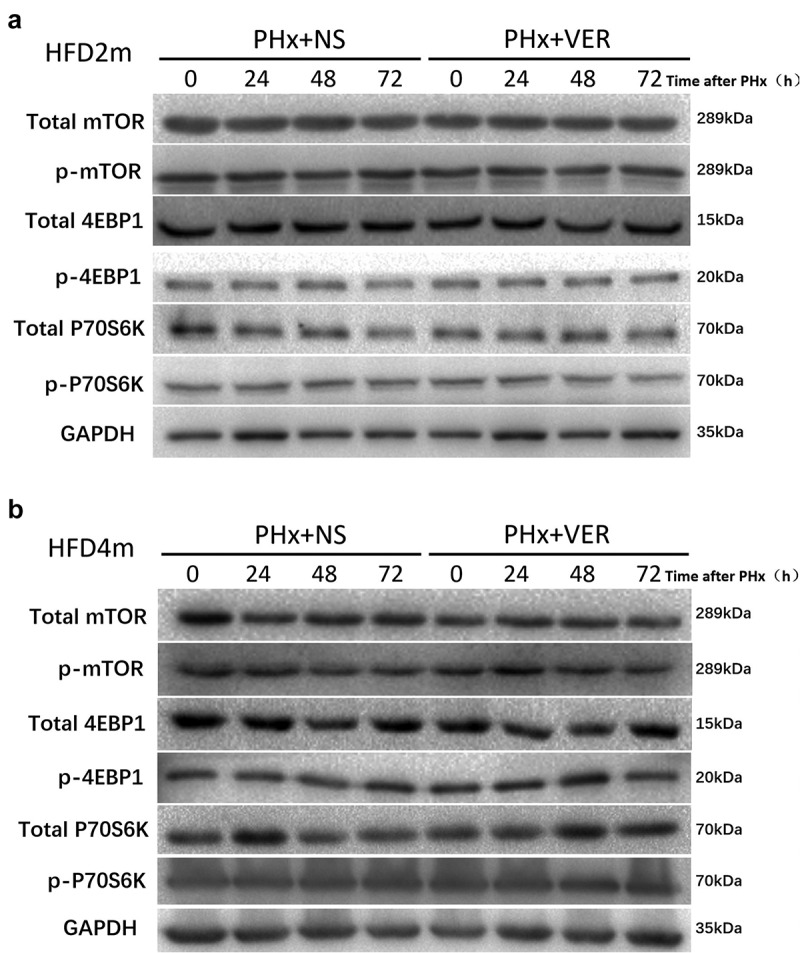
Detection of mTOR signal pathway protein after verapamil injection. a: The expression of the mTOR signal pathway protein in liver tissue of mice with mild to a moderately fatty liver after PH; b: Expression of the severe fatty liver’s mTOR signal pathway protein after PH

We further studied whether verapamil induced autophagy through the mTOR independent pathway by detecting the mTOR signal pathway’s specific proteins. In the HFD2m and HFD4m groups, there was no difference in the expression of mTOR total protein, phosphorylated mTOR, 4-EBP1 total protein, phosphorylated 4-EBP1, P70S6K total protein, and phosphorylated P70S6K protein between the verapamil and normal saline groups at 0, 24, 48, and 72 h after PH (Figure 7(a,b), Supplementary Figure S2), indicating that verapamil induces autophagy through an mTOR independent signal pathway.

## 4. Discussion

NAFLD is a kind of metabolic stress liver injury closely related to insulin resistance and genetic susceptibility.The disease spectrum includes non-alcoholic liver steatosis, non-alcoholic steatohepatitis, liver cirrhosis, and hepatocellular carcinoma [14,15]. NAFLD can lead to liver disease, disability, and death and be closely related to type 2 diabetes mellitus (T2DM), metabolic syndrome (MetS), colorectal tumour, and arteriosclerotic cardiovascular disease. With the prevalence of obesity and MetS, NAFLD has become the leading cause of chronic liver disease globally [16] and the leading cause of liver biochemical abnormalities in China [17]. When liver steatosis occurs, it will increase liver surgical complications and mortality, which is an independent risk factor for hepatectomy prognosis. The liver has a strong regeneration ability, and when damaged, it can quickly enter the cell cycle for regeneration. Hepatic steatosis reduces the hepatocytes’ response to regenerative stimuli and weakens the liver’s ability to endure ischaemia and anoxia, which harms liver regeneration [18]. However, there is no conclusion on the degree and mechanism of different causes and degrees of steatosis on liver regeneration. Hepatic steatosis can inhibit liver regeneration by regulating the endoplasmic reticulum stress signal pathway, increasing the expression of transforming growth factor B1, JAK/STAT signal pathway, ERK/MAPK signal pathway, and reduced ATP synthesis, and can also promote liver regeneration through a high expression of proliferation and anti-apoptotic molecules. We found that the control group reached the peak of proliferation at 24–48 h after PH and almost completed the process of liver regeneration at 7 d after hepatectomy.

In addition, the postoperative liver regeneration level in the fatty liver group was weaker than that in the control group, and the peak period of liver regeneration was delayed. We observed that the more severe fatty liver, the weaker liver regeneration level after hepatectomy, especially in steatohepatitis, the postoperative liver regeneration was significantly weakened and delayed to reach the peak of proliferation 48–72 h after hepatectomy. Therefore, for clinical patients with mild to moderate fatty liver, postoperative abnormal liver function should be vigilant during the perioperative period. During the perioperative period, We should actively protect the liver during the operation to improve liver regeneration damage. Hepatectomy should be avoided in the acute stage of steatohepatitis to avoid the risk of postoperative liver failure.

Autophagy is a protective response of cells to internal and external environmental stimuli. Cells can provide energy and material materials for themselves through autophagy, phagocytosis, and some organelles’ digestion to maintain cells’ normal life activities [11,19]. Autophagy plays an important role in the progression of NAFLD. Autophagy is activated in the early stage of the disease, which can delay NAFLD progression by inhibiting the ‘second blow’ of NAFLD, reducing hepatic steatosis, endoplasmic reticulum stress, inflammatory reaction etc [20–22]. In the late stage of NAFLD, autophagy was weakened due to the degradation of autophagy-related gene Atg7, the change of lipid composition of autophagy membrane and lysosome membrane, the weakening of autophagy-lysosomal proteolysis function, hyperinsulinemia, and the increase of intracellular calcium level in hepatocytes, which aggravated NAFLD [23]. Besides, interference with autophagy genes or autophagy inhibition by chloroquine exacerbated NAFLD disease progression [24], while rapamycin activation of autophagy alleviated NAFLD [21,25]. This study confirmed that although the autophagy level of moderate and severe fatty liver is increased, the overall autophagy flux process is impaired due to the increased level of LC3 and p62. At this stage, autophagy, as an adaptive response of damaged cells, removes damaged mitochondria and endoplasmic reticulum and other organelles to reduce injury and inflammation, maintain the stability of the intracellular environment, and show a protective effect. However, autophagy is damaged when NASH occurs.

At present, it has been confirmed that there is a close relationship between autophagy and liver regeneration [26]. Autophagy can coordinate with other pathways to accurately regulate liver regeneration, avoid the retardation of liver regeneration and prevent liver regeneration from exceeding the normal range. It is of great significance for maintaining the volume and function of the liver. Researchers have studied the effect of autophagy on liver regeneration after major hepatectomy in animals in recent years. In the early stage of liver regeneration, the liver needs to repair damaged hepatocytes and enter the regeneration stage, so the liver load in the early stage of liver regeneration is huge. Autophagy can help hepatocytes pass through acute stress smoothly by improving hepatocytes’ oxidative stress, reducing liver anabolism, and preserving cellular energy and nutrients. Therefore, in fatty liver mice, autophagy induced by verapamil can reduce lipid peroxidation, enhance antioxidant capacity, improve hepatocytes’ oxidative stress, and thus improve liver function.

At the same time, liver regenerative cytokines can also regulate liver regeneration through autophagy. Hepatocyte growth factor (HGF) stimulates the PI3K/Akt signal pathway to make hepatocytes from the G1 phase to the S phase. The interaction between PI3K/Akt and mTOR affects protein synthesis and cell proliferation [27]. Toshima et al.proved that autophagy plays an important role in liver regeneration after hepatectomy in normal mice [28]. In the 70% hepatectomy model of normal mice, the autophagy level increased with liver regeneration and decreased with with regeneration cessation. After knockout of autophagy gene Atg5, the ability of liver regeneration was significantly weaker than that of the control group, accompanied by increased liver volume compensation, weakening of hepatocyte mitosis, mitochondrial damage, decreased ATP production, disturbance of β-oxidation process, cell ageing, etc.

We found that the autophagy level (LC3II/I, Beclin1, Atg7) of severe fatty liver generally increased in the resting state, which was higher than that of the mild to a moderately fatty liver and normal group. The autophagy level of the steatohepatitis group was different, and the overall autophagy level of the steatohepatitis group was stronger than that of the control group, which is consistent with the results of other studies [29,30]. After hepatectomy, the autophagy level increased with the liver’s regeneration and decreased with the cessation of regeneration. The proliferation peak appeared in the control group and mild to themoderately fatty liver group at 24–48 h after an operation, while autophagy peaked at 48 h and then decreased. In the severe fatty liver group, the proliferation peak appeared at 48–72 h after PH, while autophagy reached the peak at 72 h and then decreased. This phenomenon was consistent with the theory of Toshima [28], and it also showed that there was a close relationship between autophagy and liver regeneration. The level of hepatocyte autophagy was decreased along with the gradual decrease of hepatocyte proliferation until it stopped. The results showed that autophagy-related proteins’ expression increased and the co-localized expression of LC3 and LAMP1 increased, indicating the increase of autophagy-lysosome formation and autophagy flux, which represents the dynamic process of autophagy from cargo sequestration to its degradation. The lysosome is the last step of autophagy flux, so the lysosomal function is crucial for completing the whole process of autophagy.

The mTOR is a key regulator of autophagy and cell proliferation [31]. Autophagy can be activated in the mTOR-dependent and mTOR-independent signalling pathways. However, the mTOR pathway is a key regulatory pathway of autophagy and regulating cell proliferation. Inhibition of mTOR activity can induce autophagy and inhibit cell proliferation, such as rapamycin [32], sirolimus, and so on. In the case of hepatectomy, inhibiting cell proliferation is harmful, so mTOR-dependent autophagy inducers are not suitable for promoting liver regeneration. Verapamil is a widely used antiarrhythmic drug, which can reduce calcium permeability and increase potassium permeability. It can also induce autophagy through the mTOR-independent signalling pathway [13]. Recent studies have found that verapamil can inhibit the expression of TXNIP in islet P cells, improve islet P cell function, promote insulin synthesis and release, and improve insulin resistance and glucose metabolism disorder in diabetic mice [33]. The study also found that verapamil can inhibit inflammation in the liver of obese mice [12].

Therefore, to test the hypothesis that verapamil can enhance liver regeneration through autophagy, we used verapamil to induce autophagy in mice receiving PH. We found that in PH mice treated with verapamil, autophagy was activated and enhanced before and after an operation. The body weight, blood glucose, and transaminase of the mild, moderate, and severe fatty liver mice decreased and lipid infiltration reduced, but the antioxidant capacity enhanced and liver function improved. After PH, autophagy was observed to increase, which also promoted the process of liver regeneration. We found that verapamil did not affect autophagy through the mTOR signal pathway by detecting the related classical proteins. Therefore, verapamil can up-regulate autophagy flux through independent of mTOR signalling pathway, so as to induce beneficial hepatocyte proliferation and liver growth. Indeed, verapamil enhancing autophagy’s pharmacological effects may be a new strategy to promote liver regeneration, hepatocyte proliferation and survival. As far as we know, this discovery has not been reported in the previous literature.

## 5. Conclusions

To sum up, we proved that autophagy plays a key role in promoting liver regeneration, alleviating liver injury, and prolonging mice’s survival after PH. The pharmacological regulation of verapamil on autophagy effectively promoted liver regeneration, hepatocyte production, and survival in mice and reduced liver injury in mice after PH. Autophagy is expected to become a key target for NAFLD treatment and open up a new direction for the prevention and treatment of obesity, T2DM, Mets, and other diseases.

## Supplementary Material

Supplemental Material

## Data Availability

The data that support the findings of this study are available in ‘4TU. ResearchData’ at https://data.4tu.nl/authors/JianLin_Lai/11433763.
