# Supplementary material for: Verapamil induces autophagy to improve liver regeneration in non-alcoholic fatty liver mice
Source: Adipocyte. 2021 Oct 26;10(1):532–45. doi: 10.1080/21623945.2021.1983241 (PMC8555529; doi:10.1080/21623945.2021.1983241)
Supplement: Supplemental Material [file KADI_A_1983241_SM3986.zip › supplementary/Supplementary Fig legends.docx]

Supplementary Fig. 1. Autophagy-related genes (LC3/p62/Beclin1/Atg7) mRNA levels were measured by RT-qPCR in corresponding liver samples in the control group (ND4m), mild to moderately fatty liver group (HFD2m), severe fatty liver group (HFD4m), and fatty hepatitis group (HFD+CCl4) at rest and after hepatectomy. A: The mRNA levels of LC3/p62/Beclin1/Atg7 at resting state; B: q-PCR to detect the changing trend of LC3 mRNA level after hepatectomy; C: q-PCR to detect the changing trend of p62 mRNA level after hepatectomy; D: q-PCR to detect the changing trend of Beclin1 mRNA level after hepatectomy; E: q-PCR to detect the changing trend of Atg7 mRNA level after hepatectomy. (*: 0.01<P<0.05, **: 0.001<P<0.01, ***: 0.0001<P<0.001, ****: P<0.0001, n=10.)

Supplementary Fig. 2. The protein levels of mTOR, p-mTOR, 4EBP1, p-4EBP1, P70S6K, p-P70S6K in total liver after VER rejection and hepatectomy were assessed by western blot, quantified using ImageJ analysis and normalized to GAPDH. A: The relative intensity of p-mTOR/mTOR protein in liver tissue of mice with mild to a moderately fatty liver; B: The relative intensity of p-4EBP1/4EBP1 protein in liver tissue of mice with mild to a moderately fatty liver; C: The relative intensity of p-P70S6K/P70S6K protein in liver tissue of mice with mild to a moderately fatty liver; D: The relative intensity of p-mTOR/mTOR protein in liver tissue of mice with severe fatty liver; E: The relative intensity of p-4EBP1/4EBP1 protein in liver tissue of mice with severe fatty liver; F: The relative intensity of p-P70S6K/P70S6K protein in liver tissue of mice with severe fatty liver.
