# Supplementary figures and images for: Verapamil induces autophagy to improve liver regeneration in non-alcoholic fatty liver mice
Source: Adipocyte. 2021 Oct 26;10(1):532–45. doi: 10.1080/21623945.2021.1983241 (PMC8555529; doi:10.1080/21623945.2021.1983241)

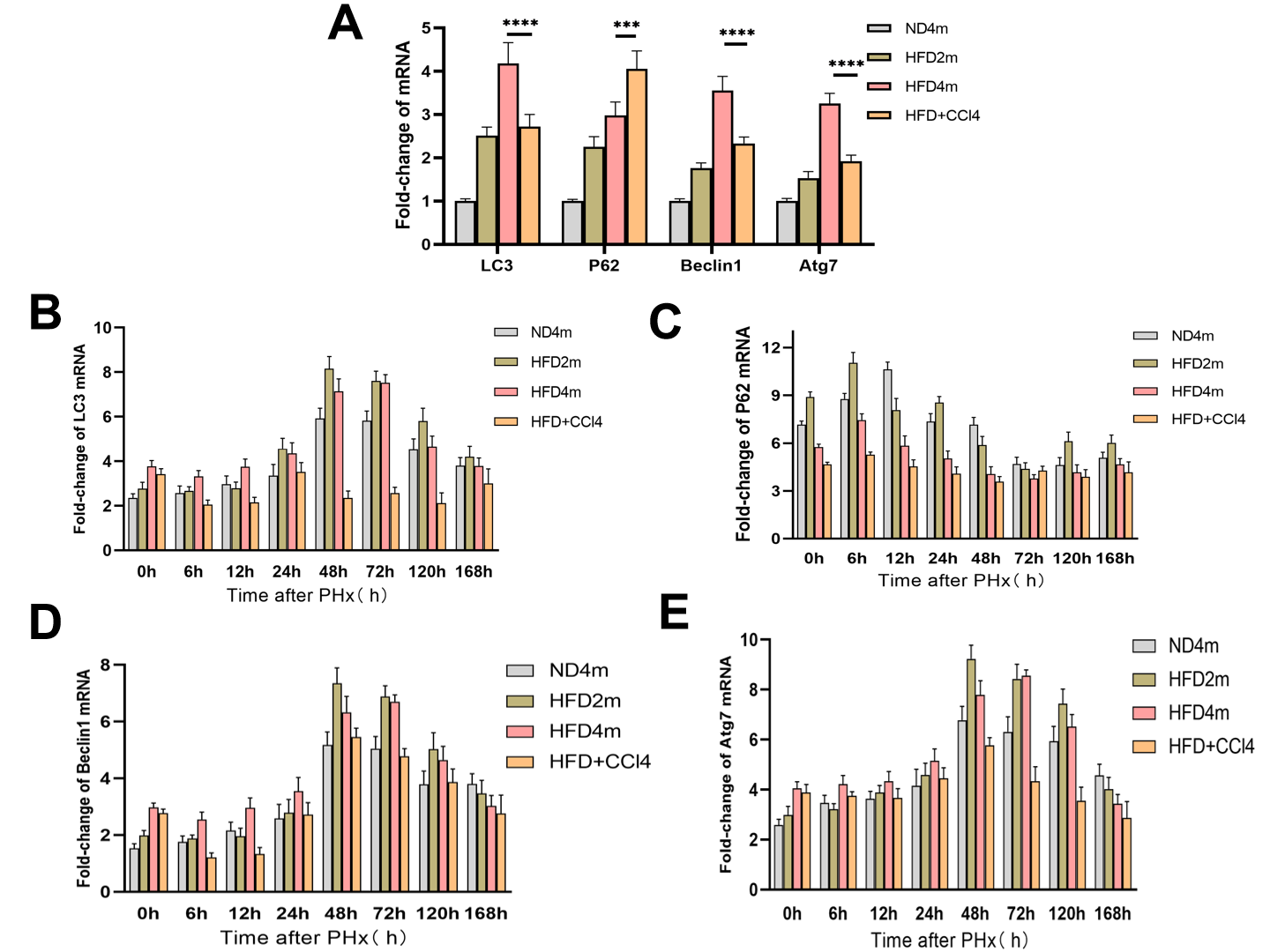

Supplement: Supplemental Material [file KADI_A_1983241_SM3986.zip › supplementary/Supplementary Figure 1 (3).tif]

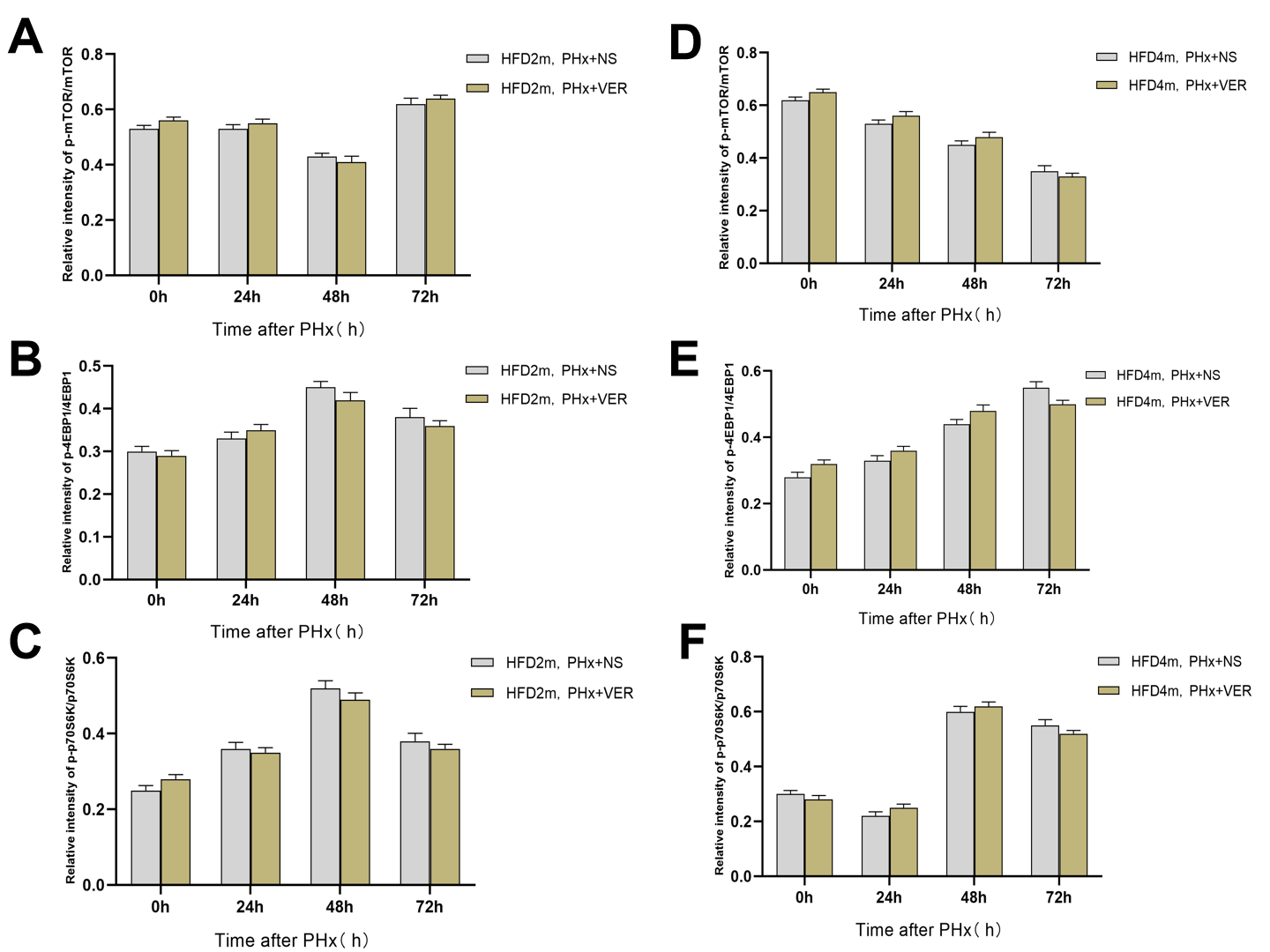

Supplement: Supplemental Material [file KADI_A_1983241_SM3986.zip › supplementary/Supplementary Figure 2 (1).tif]
